# Recent Progress of Protein Tertiary Structure Prediction

**DOI:** 10.3390/molecules29040832

**Published:** 2024-02-13

**Authors:** Qiqige Wuyun, Yihan Chen, Yifeng Shen, Yang Cao, Gang Hu, Wei Cui, Jianzhao Gao, Wei Zheng

**Affiliations:** 1 Department of Computer Science and Engineering, Michigan State University, East Lansing, MI 48824, USA; 2 School of Mathematical Sciences and LPMC, Nankai University, Tianjin 300071, China; yh_chen@mail.nankai.edu.cn; 3 Faculty of Environment and Information Studies, Keio University, Fujisawa 252-0882, Kanagawa, Japan; tomshen@keio.jp; 4 College of Life Sciences, Sichuan University, Chengdu 610065, China; 5 NITFID, School of Statistics and Data Science, LPMC and KLMDASR, Nankai University, Tianjin 300071, China; 6 Department of Computational Medicine and Bioinformatics, University of Michigan, Ann Arbor, MI 48109, USA

**Keywords:** AlphaFold2, contact map, deep learning, distance map, end-to-end methods, multi-domain proteins, protein language model, protein tertiary structure prediction, template-based modeling, template-free modeling

## Abstract

The prediction of three-dimensional (3D) protein structure from amino acid sequences has stood as a significant challenge in computational and structural bioinformatics for decades. Recently, the widespread integration of artificial intelligence (AI) algorithms has substantially expedited advancements in protein structure prediction, yielding numerous significant milestones. In particular, the end-to-end deep learning method AlphaFold2 has facilitated the rise of structure prediction performance to new heights, regularly competitive with experimental structures in the 14th Critical Assessment of Protein Structure Prediction (CASP14). To provide a comprehensive understanding and guide future research in the field of protein structure prediction for researchers, this review describes various methodologies, assessments, and databases in protein structure prediction, including traditionally used protein structure prediction methods, such as template-based modeling (TBM) and template-free modeling (FM) approaches; recently developed deep learning-based methods, such as contact/distance-guided methods, end-to-end folding methods, and protein language model (PLM)-based methods; multi-domain protein structure prediction methods; the CASP experiments and related assessments; and the recently released AlphaFold Protein Structure Database (AlphaFold DB). We discuss their advantages, disadvantages, and application scopes, aiming to provide researchers with insights through which to understand the limitations, contexts, and effective selections of protein structure prediction methods in protein-related fields.

## 1. Introduction

Proteins are macromolecules that play important roles in facilitating the essential functions vital for life’s sustenance. Their pivotal involvement spans a diverse array—providing structural support to cells, safeguarding the immune system, catalyzing crucial enzymatic reactions, orchestrating cellular signal transmission, regulating the intricate processes of transcription and translation, and encompassing the synthesis and breakdown of biomolecules. Moreover, they contribute significantly to the regulation of developmental processes, biological pathways, and the constitution of protein complexes and subcellular structures. These diverse and remarkable functions originate from their distinct three-dimensional (3D) structures, which vary across different protein molecules. Since Anfinsen showed that the tertiary structure of a protein is determined by its amino acid sequence in 1973 [1], understanding the protein sequence–structure–function paradigm has emerged as a fundamental cornerstone within modern biomedical studies. Due to significant efforts in genome sequencing over the last few decades [2,3,4], the number of known amino acid sequences deposited in UniProt [5] has grown to over 250 million. Despite the impressive number of data, the amino acid sequences themselves only offer limited insights into the biological functions of individual proteins, as these functions are primarily determined by their three-dimensional structures.

Some of the most widely used experimental techniques for determining protein structures include X-ray crystallography [6], NMR spectroscopy [7], and cryo-electron microscopy [8]. Despite their accuracy, the considerable human involvement and substantial expenses involved in experimentally resolving a protein’s structure have hindered advancement in the number of solved protein structures. Consequently, the expansion in solved protein structures has considerably trailed the accumulation of protein sequences. At present, the Protein Data Bank [9] (PDB) contains structures for approximately 0.21 million proteins, accounting for less than 0.1% of the total sequences cataloged in the UniProt database [10]. This disparity highlights the ever-widening gap between known protein sequences and experimentally solved protein structures. Nevertheless, owing to substantial collective efforts within the scientific community in recent decades [11,12,13,14,15,16,17,18,19,20,21,22,23,24,25], computational approaches have made remarkable progress, through which an increasing fraction of sequences in various organisms have had their tertiary structures reliably modeled [26,27,28,29,30,31,32,33,34,35,36,37,38,39]. For example, the first version of AlphaFold demonstrated exceptional predictive capabilities in protein structure prediction by employing the deep learning-based distance map prediction during the 13th Critical Assessment of Protein Structure Prediction (CASP13). Furthermore, with the utilization of the end-to-end deep learning approach, the AlphaFold2 has facilitated the rise of structure prediction performance to new heights, regularly competitive with experimental structures in CASP14. These methodologies have significantly contributed to diverse biomedical investigations, including structure-based protein function annotation [40,41,42,43,44], mutation analysis [45,46,47,48,49,50,51,52], ligand screening [53,54,55,56,57,58,59], and drug discovery [60,61,62,63,64,65].

In this review, we start with an overview of the history of protein structure prediction, including template-based modeling (TBM) and template-free modeling (FM) methods. TBM techniques predict models by refining the structures of existing proteins, known as templates, identified from the PDB. In contrast, FM methods construct protein structures without relying on template structures. Then, we discuss the recent advancements and progress brought about by deep learning technologies, including contact/distance-guided protein structure prediction methods, end-to-end folding methods, and protein language model (PLM)-based methods. In particular, we highlight the breakthrough in end-to-end methods and protein language model (PLM)-based methods. Additionally, we introduce recent progress in multi-domain protein structure predictions. Finally, we describe the CASP experiments and some widely used assessment measures for protein structure prediction, followed by the introduction of the recently released AlphaFold Protein Structure Database (AlphaFold DB) and its corresponding applications.

**Figure 1:**
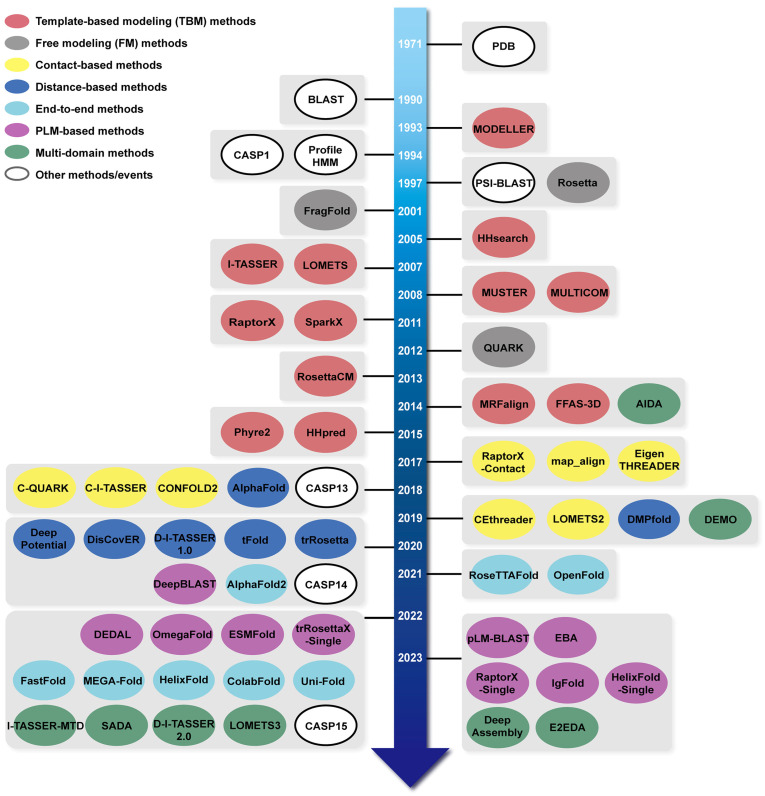
The timeline of important methods or tools in protein structure prediction. Different methods or tools are denoted by different colors: template-based modelling (TBM) methods are represented by red, free modeling (FM) methods by gray, contact-based methods by yellow, distance-based methods by blue, end-to-end-based methods by cyan, protein language model (PLM)-based methods by purple, and multi-domain methods by green, while other important methods or events are highlighted in white. Note that some methods may be categorized under two or more groups, but we only highlighted the most important category for each method.

Tables S1–S7 offer links to the methods discussed in this review, serving as a supplemental resource for readers’ accessibility. Meanwhile, Figure 1 presents a comprehensive timeline of these methods and some significant achievements covered in this review.

## 2. An Overview of Protein Structure Prediction

### 2.1. Template-Based Modeling (TBM) Methods

**Figure 2:**
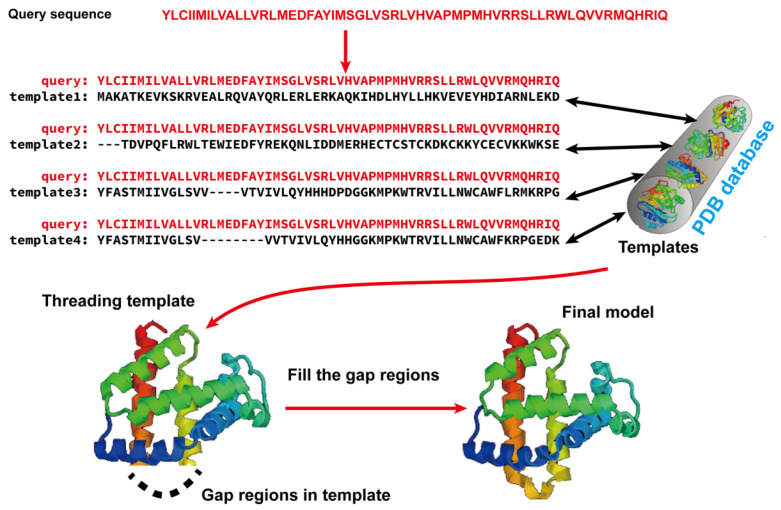
Illustration of template-based modeling (TBM) methods. Starting from a query sequence, templates are identified from Protein Data Bank (PDB) and subsequently aligned with the query protein sequence. Then, the final structural model is constructed by replicating the aligned regions and refining the unaligned regions.

Template-based modeling (TBM) methods have emerged as pivotal approaches in the realm of computational biology for predicting protein structures. TBM leverages known protein structures, referred to as templates, from the PDB to predict the structure of an unknown protein (target), assuming that the target shares a significant degree of sequence similarity with the template. As shown in Figure 2, TBM methods usually consist of the following four steps: (i) identifying templates related to the protein of interest, (ii) aligning the query protein with the templates, (iii) building the initial structural framework by replicating the aligned regions, and (iv) constructing the unaligned regions and refining the structure. TBM can be classified as homology modeling (comparative modeling), which is often employed when there is substantial sequence identity—typically 30% or greater—between the template and the protein of interest, and threading (fold recognition), which is used when the sequence identity drops below the 30% threshold [66].

In homology modeling, high-quality templates are detected and aligned using straightforward sequence–sequence alignment algorithms, such as dynamic programming-based techniques like the Needleman–Wunsch [67] algorithm for global alignment and the Smith–Waterman [68] algorithm for local alignment. BLAST [69] is another widely used tool to identify templates and generate alignments, which initially identified short matches between the query and template, and then extended these matches to generate alignments.

In threading, since the sequence identity between the best available template and the query protein falls below 30%, it is hard to identify templates simply based on straightforward sequence–sequence alignment algorithms. Hence, the 1D profile of local structural features is used to represent a template’s 3D structure, because they are often more conserved than the amino acid identities themselves and, thus, can be used to identify and align proteins with similar structures but more distant sequence homology. A commonly used sequence profile is the Position-specific Scoring Matrix (PSSM), which captures the amino acid tendencies at each position within the multiple sequence alignment (MSA). The PSSM is iteratively employed to search through a template database, aiming to identify distantly homologous templates for a specific protein sequence. One popularly used profile-based threading algorithm is MUSTER [70], which combines various sequence and structural information into single-body terms in a dynamic programming search, as follows: (i) sequence profiles; (ii) secondary structures; (iii) structure fragment profiles; (iv) solvent accessibility; (v) dihedral torsion angles; and (vi) hydrophobic scoring matrix. In addition to PSSMs, profile hidden Markov models (HMMs) are another type of sequence profile. A profile HMM is a probabilistic model that captures the evolutionary changes within an MSA. The key advantage of profile HMMs lies in their utilization of position-specific gap penalties and substitution probabilities, providing a closer representation of the true underlying sequence distribution [71]. HHsearch [72] is the most widely used profile HMM-based threading method, which generalized the alignment of protein sequences with a profile HMM to the case of pairwise alignment of profile HMMs for detecting distant homologous relationships between proteins.

Given the recent substantial improvements in contact and distance map prediction using deep learning, which will be discussed later, threading methods guided by these maps represent the cutting edge in fold recognition, achieving superior accuracy compared to general profile or profile HMM-based threading methods. Among these approaches, EigenTHREADER [73] utilized the eigen decomposition of contact maps to derive the primary eigenvectors, which were used for aligning the template and query contact maps. CEthreader [74], employing a similar eigen decomposition strategy, outperformed pure contact map-based threading methods by integrating data from local structural feature prediction and sequence-based profiles. map_align [21], on the other hand, introduced an iterative dual dynamic programming technique to align contact maps, while DeepThreader [75] leveraged predicted distance maps to establish alignments. Most recently, DisCovER [76] integrated deep learning-predicted distance and orientation into the threading method by generating alignments through an iterative double dynamic programming framework. In addition, meta-threading approaches, such as LOMETS [77,78,79], combine the templates’ output, via multiple threading programs, into a set of consensus templates, thereby attaining enhanced accuracy. For example, LOMETS2 [78] integrated a comprehensive set of state-of-the-art threading programs, including contact-guided threading approaches, and utilizes deep profiles generated by a novel deep MSA construction method, DeepMSA [80].

Furthermore, deep learning-based methods have been directly applied to recognize distant homology templates. The cutting-edge methods, such as ThreaderAI [81] and SAdLSA [82], conceptualize the task of aligning query sequence with template as the classical pixel classification problem in computer vision, which allows for the integration of a deep residual neural network [83] into fold recognition. More recently, the application of language models, originally developed for text classification and generative tasks, to protein sequences marks a significant advancement in the bioinformatics field. Protein language models (PLMs) are a type of neural network with self-supervised training on an extensive number of protein sequences [84,85]. Once trained, PLMs can be used to rapidly generate high-dimensional embeddings on a per-residue level, which can be viewed as a “semantic meaning” of each amino acid within the context of the full protein sequence. Such representations have proven invaluable in identifying distant homologous relationships between proteins. For example, pLM-BLAST [86] detected distant homologous relationships by integrating single-sequence embeddings, obtained from protein language models (PLMs), with a local similarity detection algorithm from BLAST. pLM-BLAST operated on an unsupervised basis, eliminating the need for training a specialized deep-learning model, and was capable of computing both local and global alignments, leveraging the strengths of PLM-derived embeddings and BLAST-based algorithms. EBA [87] was a new tool designed to generate embedding-based protein sequence alignments, particularly in the challenging ‘twilight zone’. It leveraged the distances between all possible pairs of residue embeddings to create a “similarity matrix.” This matrix subsequently served as a scoring matrix within a classical dynamic programming alignment framework. The absence of any requirement for training and parameter optimization, coupled with its flexibility to any language model, rendered the EBA method robust to generalization and easy to interpret. DEDAL [88] and DeepBLAST [89] both integrated residue embeddings learned from a PLM into a differentiable alignment framework; however, DEDAL used an affine scoring function, while DeepBLAST had a simpler linear model for scores and only produced global alignments. Due to their rich information contents, sequence embeddings produced by PLMs have been successfully applied to many other tasks, especially in the prediction of tertiary structures, which will be discussed later.

Once the templates are identified and aligned with the query proteins, the subsequent step involves building a model by replicating and refining the structure of the template. The most widely used method was MODELLER [16], which constructed tertiary structure models by optimally satisfying spatial constraints extracted from the template alignments, along with other general structural constraints, such as ideal bond lengths, bond angles, and dihedral angles. Furthermore, the new HHpred modeling pipeline, proposed by the Söding group, has extended the MODELLER by employing (i) atomic distance restraints described by two-component Gaussian mixtures, (ii) optimal weights to correct for redundancy among related templates, and (iii) a heuristic template selection strategy [90].

With the development of computational techniques, some methods are proposed to convert alignments directly into 3D models. A notable example is I-TASSER [91,92,93], an extension of TASSER [28]. This method utilized a process wherein continuous fragments were extracted from the aligned regions of multiple threading templates identified by LOMETS. These fragments were reassembled during structure assembly simulations. I-TASSER incorporated constraints derived from template alignments and a set of knowledge-based energy terms. These energy terms included hydrogen bonding, secondary structure formation, and side-chain contact formation. The integration of these components was used to guide the Replica Exchange Monte Carlo (REMC) simulation. After clustering low-energy decoys and selecting the centroid of the most favorable cluster, the centroid was compared against the PDB to identify additional templates. The constraints from these new templates, combined with those from the initial cluster model and threading templates, as well as the intrinsic knowledge-based potentials, were employed to direct a subsequent round of structure assembly simulations. The lowest energy structure was selected, which was then subjected to full-atom refinement. Since its first emergence in the CASP7, I-TASSER has consistently achieved top rankings among automated protein structure prediction servers in subsequent CASP experiments [66]. Another example is RosettaCM [94], that assembled structures using integrated torsion space-based and Cartesian space template fragment recombination, loop closure by iterative fragment assembly and Cartesian space minimization, and high-resolution refinement.

### 2.2. Fragment Assembly Simulation Methods for Free Modeling (FM)

Theoretically, all-atom molecular dynamics (MD) simulations are able to predict protein structures if the computer is powerful enough. However, modern MD simulations can only deal with proteins of less than ~100 amino acids in size. Thus, 90% of the natural proteins cannot be predicted because of the required computational complexity [95]. Hence, an alternative method, namely free modeling (FM), was proposed to model protein structures. Compared to MD simulations, FM methods employ the coarse-grained protein elements and physics- or knowledge-based energy functions, together with extensive sampling procedures, to construct protein structure models from scratch. In contrast to TBM methods, they do not depend on global templates. Hence, they are commonly referred to as ab initio or de novo modeling approaches [17,19]. Since the nature of coarse-grained protein leads to inherent inaccuracies, FM methods, historically, have not achieved levels of accuracy comparable to those of TBM methods, if the global templates are available.

**Figure 3:**
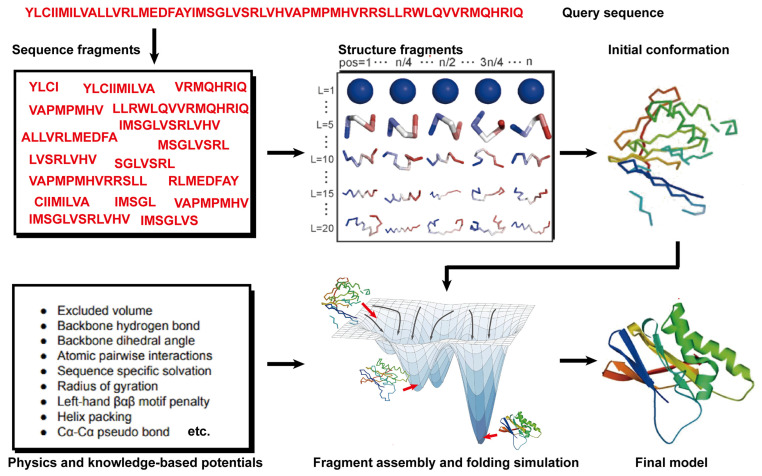
Illustration of free modeling (FM) methods. Starting from a query sequence, local fragments are identified from databases of solved protein structures, using profile-based threading methods. These fragments are subsequently utilized to construct full-length structural models, guided by physics- or knowledge-based energy potentials.

State-of-the-art FM methods have evolved to assemble protein fragments [96]. These fragment assembly techniques assume that protein fragments extracted from the PDB covered most of the conformation of protein folding. Thus, the sampling space was sharply narrowed down. Their implementation involves generating a set of fixed-length (9 residues) and variable-length (15–25 residues) fragments from a repository of known 3D structures (as shown in Figure 3). These fragments are subsequently linked, rotated, and scored to find the global minimum state. This methodology of fragment assembly serves to reduce the exploration of conformational space while ensuring the coherent formation of local structures within the assembled fragments.

The first version of Rosetta modeling software, released in 1997, is one of the most well-known FM methods developed by David Baker’s group [17]. Rosetta utilized a three- and nine-residue fragment database for assembly. Particularly, the fragments were selected by quantifying the profile–profile and secondary structure similarity between the query sequence and fragment database within a defined window size. The fragments were simplified to backbone atoms and side-chain centers, and subsequently conducted by simulated annealing Monte Carlo simulations, which exchanged the backbone torsion angles with those of one of the highly scored fragments in the database. A centroid energy function was utilized to guide the simulation, incorporating various factors, such as helix-strand packing, strand pairing, solvation, van der Waals interactions, the radius of gyration, the arrangement of strands into sheets, and interactions between residue pairs. Conformations that exhibited favorable local interactions and possessed protein-like global properties during the simulation were clustered based on their structural similarity, and the final structure was obtained from the center of the largest cluster.

QUARK is another state-of-the-art FM method developed by Yang Zhang’s group [19]. Unlike the conventional fragment assembly methods, QUARK utilized distinct methodologies for fragment generation and energy function design. It integrated a distance-based profile energy term, estimating and restricting the distance between two residues by considering inter-residue distances from fragments sourced from the same PDB structures. Additionally, QUARK incorporated 11 diverse conformational movements, improving the efficiency of the conformational sampling procedure, alongside the fragment replacement movement. Today, both the QUARK and Rosetta methods have achieved levels of accuracy comparable to those of TBM methods, and are particularly useful when the protein templates are not available.

### 2.3. Contact-Based Protein Structure Prediction

A contact map for a protein of length *L* is defined as a symmetric, binary *L* × *L* matrix. Each element in the matrix represents a binary value, signifying whether the residues form a contact (Cβ-Cβ distance (Cα for glycine) < 8 Å) or not. Since the concept of contact was first brought up, many attempts were made to predict contacts based on correlated mutations in MSAs [97,98,99]. The hypothesis behind these approaches was that residue pairs that are in contact in 3D space would exhibit correlated mutation patterns, also known as co-evolution (Figure S1), because there is evolutionary pressure to conserve the structures of proteins. A widely used type among these methods is the direct coupling analysis (DCA) method, which considers the full set of pairwise interactions instead of evaluating residues individually. This approach has obtained improved performance compared to mutual information-based methods [99].

In the early 2010s, an increasing number of predictors began integrating deep learning architectures into their prediction methods. A breakthrough occurred in 2017, when Xu’s group introduced RaptorX-Contact [22], which revolutionized contact prediction by integrating deep residual convolutional neural networks (ResNets [83]). A Residual Neural Network incorporates an identity map of the input to the output of the convolutional layer, facilitating smoother gradient flow from deeper to shallower layers and enabling training of deep networks with numerous layers. RaptorX-Contact’s utilization of deep ResNets, featuring approximately 60 hidden layers, led to a significant performance leap, outstripping other methods [66]. The introduction of deep ResNets, consisting of approximately 60 hidden layers, enabled RaptorX-Contact to significantly outperform other methods [66]. Following RaptorX-Contact’s paradigm, several similar methods, like TripletRes [100,101], have emerged.

**Figure 4:**
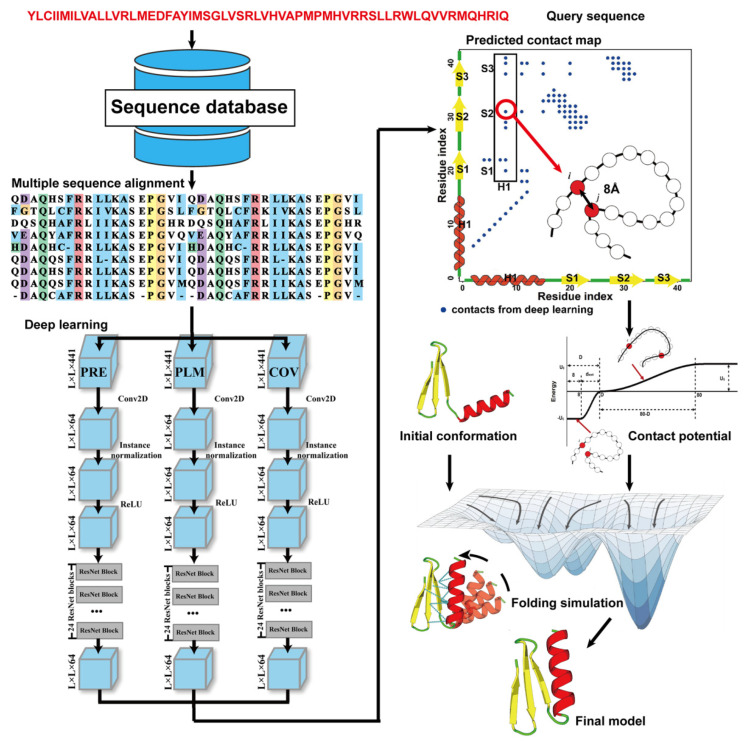
Illustration of contact-based protein structure prediction methods. Starting from a query sequence, an MSA is first generated by searching through databases. The MSA is then used as the input of deep learning methods to predict a contact map. Finally, the contact potential derived from the predicted contact map is used in a folding simulation to predict the final model.

Due to the latest advances in residue–residue contact prediction, contact-guided protein structure prediction methods have been developed and are becoming more and more successful. The idea of contact-based protein structure prediction methods is described in Figure 4. Starting from a query sequence, an MSA is first generated by searching through databases. The MSA is then used as the input for deep learning methods to predict a contact map. Finally, the contact potential derived from the predicted contact map is used in a folding simulation to predict the final model.

An example of contact-based protein structure prediction methods is CONFOLD2 [102], which builds models using various subsets of input contacts to explore the fold space under the guidance of a soft square energy function, and then clusters the models to obtain the top five models. 

The efficacy of deep learning-based contact map prediction was clearly demonstrated by C-I-TASSER and C-QUARK during CASP13, where they ranked in the top two positions among automated servers [23]. These two servers, extended from the classic I-TASSER and QUARK frameworks, incorporated contact maps derived from TripletRes [100,101], ResPRE [103], and various deep learning-based predictors into their simulations. The inclusion of these deep learning restraints significantly enhanced modeling accuracy, particularly for targets lacking easily identifiable template structures [23].

### 2.4. Distance-Based Protein Structure Prediction

**Figure 5:**
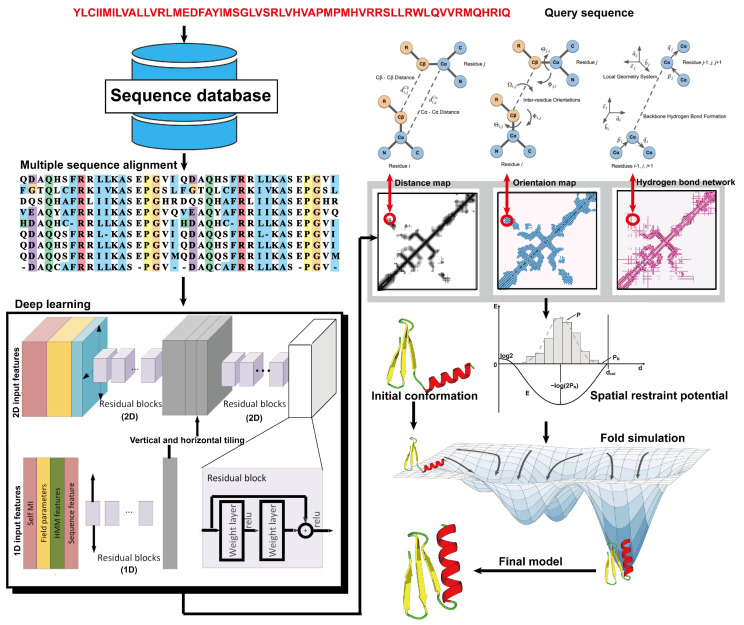
Illustration of distance-based protein structure prediction methods. Starting from a query sequence, an MSA is first generated by searching through databases. Then, the MSA is fed into deep neural networks to predict spatial restraints, such as distance maps, inter-residue orientations, and hydrogen bond networks. Finally, the final structural model is constructed by employing the potentials extracted from the predicted spatial restraints in a folding simulation to identify the lowest energy structure.

From the definition of contact map prediction, a more detailed extension is distance map prediction. The distinction lies in contact map prediction entailing binary classification, whereas distance map prediction generally estimates the likelihood of the distance between residues falling within various bins (despite attempts made to directly predict real-value distances [104]). Distance map prediction gained significant prominence in the field during CASP13 in 2018, when RaptorX-Contact [22], DMPfold [105], and AlphaFold [106] extended the application of deep ResNets from contact prediction to distance prediction. Among these predictors, AlphaFold, created by Google DeepMind, exhibited superior performance in tertiary structure modeling, as it was ranked as the top one among all groups in CASP13. Leveraging co-evolutionary coupling information extracted from an MSA, AlphaFold employed a deep residual neural network, comprising 220 residual blocks, to predict the distance map for a target sequence, which was subsequently used to assemble protein models. Figure 5 shows the basic steps of distance-based protein structure prediction methods.

A further expansion beyond distance prediction is the prediction of inter-residue torsion angle orientations. The significance of orientation-dependent energy functions serves a dual purpose: biologically, certain residue–residue interactions necessitate not only proximity in distance but also specific orientations between the residue pairs, such as beta strand pairing. From a mathematical standpoint, the inclusion of torsion angle information is crucial, as distance data alone cannot distinctly discern between a pair of mirrored structures, rendering it impossible to uniquely determine the geometry of a structure.

Due to the significance of inter-residue orientations, numerous structure prediction methodologies have integrated them into their workflows. For instance, trRosetta [25,107] has included orientation information by employing a deep residual neural network to predict both pairwise residue distances and inter-residue orientations, based on co-evolutionary information. In CASP14, several leading groups, including D-I-TASSER [108] and D-QUARK [108], incorporated orientation and distance restraints predicted by deep residual neural networks. Moreover, the top CASP14 server group, D-I-TASSER, utilized DeepPotential’s residual neural network to predict hydrogen bond networks and integrated these hydrogen bonding restraints into its structural assembly simulations. Notably, the deep learning-based hydrogen bond network prediction significantly enhanced modeling accuracy for CASP14 targets, particularly those lacking homologous templates [108].

### 2.5. End-to-End Protein Structure Prediction

AlphaFold2 achieved remarkable modeling accuracy and substantially addressed the challenge of predicting the structures of single-domain proteins in CASP14 [109]. The success of AlphaFold2 can be attributed, in part, to its unique “end-to-end” learning approach. This end-to-end learning approach eliminates the need for complex folding simulations, allowing deep neural networks, such as 3D equivariant transformers in AlphaFold2, to predict structural models directly.

AlphaFold2 adopted a novel architecture that is quite different from those of previous methods, including the first version of AlphaFold, to accomplish end-to-end structure prediction. The architecture of AlphaFold2 includes the following two primary components: the Trunk Module, which utilizes self-attention transformers to process input data consisting of the query sequence, templates, and MSA; and the Structure (or Head) Module, which employs 3D rigid body frames to directly generate 3D structures from the training components [110].

Despite its breakthrough in accuracy and performance, AlphaFold2 has notable limitations, such as increased time consumption with longer protein lengths. To address these challenges, several faster artificial intelligence-driven protein folding tools, based on AlphaFold2, have been developed [111,112,113]. For example, ColabFold [111] improved the speed of protein structure prediction by integrating MMseqs2′s efficient homology search (Many-against-Many sequence searching) [114] with AlphaFold2 [110]. OpenFold [112], a trainable and open-source implementation of AlphaFold2 using PyTorch [115], achieved enhanced computational efficiency with reduced memory usage, thereby facilitating the prediction of exceedingly long proteins on a single GPU. Similarly, Uni-Fold [113] redeveloped AlphaFold2 within the PyTorch framework and reproduced its original training process on a larger set of training data, achieving comparable or superior accuracy and faster speed. Collectively, these developments represent significant strides in enabling rapid and accurate predictions of protein structures.

Table 1 and Table 2 show both the domain-level and full-length-level comparisons of TM-scores among AlphaFold2 and its follow-up methods on CASP14 targets (target details are shown in Table S8). The domain-level targets (or domains) are further classified as “TBM-easy”, “TBM-hard”, “FM/TBM”, or “FM” by CASP, depending on the availability and quality of PDB templates for each domain, wherein “TBM-easy” domains have readily identifiable, high-quality templates and “FM” domains typically lack homologous templates in the PDB. To simplify the analysis, “TBM-easy” and “TBM-hard” domains have been merged into “TBM” domains, and “FM/TBM” and “FM” domains into “FM” domains. Here, TM score is a sequence length-independent metric that ranges from [0, 1], in which a score >0.5 indicates that the predicted and native structures share the same global topology [116,117]. From the tables, AlphaFold2 showed excellent performance, only slightly worse than Uni-Fold and ColabFold, especially on FM targets, because of the larger number of training data (that may include CASP14 targets) used in Uni-Fold and the improved MMseqs2-based MSA construction used in ColabFold. Furthermore, AlphaFold2 had an average TM-score of 0.8871 on domain-level assessments (Table 1), but only 0.8514 on full-length-level assessments (Table 2). This is because the full-length-level assessments account for multi-domain targets, whereas AlphaFold2 still needs to be improved. Similar trends can be seen for other AlphaFold2-based methods, indicating that AlphaFold2 and its follow-up methods still need to improve their multi-domain protein structure predictions, even though they have excellent performance on single-domain proteins.

In addition to AlphaFold2 and its related methods, Baker’s group has developed RoseTTAFold [118], which used a three-track network to process sequence, distance, and coordinate information simultaneously, and achieved high prediction accuracy at CASP14, ranking only behind AlphaFold2.

### 2.6. Protein Language Model-Based Protein Structure Prediction

AlphaFold2 has facilitated the rise of structure prediction performance to new heights, nearly comparable to the accuracy of experimental determination methods since CASP14. Standard protein structure prediction pipelines heavily rely on co-evolution information from MSAs. However, the excessive dependence on MSAs often acts as a bottleneck in various protein-related problems. While model inference in the structure prediction pipeline typically takes a few seconds, the MSA construction step is time-intensive, consuming tens of minutes per protein. This time-consuming process significantly hampers tasks requiring high-throughput requests, like protein design [119]. Therefore, developing an accurate and efficient MSA-free protein structure prediction method holds promise in advancing protein studies.

A large-scale protein language model (PLM) presents an alternative avenue to MSAs for acquiring co-evolutionary knowledge, facilitating MSA-free predictions. In contrast to MSA-based methods, wherein information retrieval techniques explicitly capture co-evolutionary details from protein sequence databases, PLM-based methods embed co-evolutionary information into the large-scale model parameters during training, and allow for implicit retrieval through model inference, wherein the PLM is viewed as a repository of protein information. Furthermore, MSA-based approaches have lower efficiency in information retrieval, relying on manually designed retrieval schemes. Conversely, a PLM-based method showcases heightened efficiency in information retrieval, with retrieval quality predominantly influenced by the model’s capacity or parameter size. A lot of pre-trained PLMs have been developed and released for various downstream analyses [85,120], such as SaProt [120], which is a large-scale general-purpose PLM trained on an extensive dataset comprising approximately 40 million protein sequences and structures, and ESM-2 [85], which was trained on protein sequences from the UniRef database, with up to 15 billion parameters.

Inspired by the progress of PLMs and AlphaFold2, many protein structure prediction methods have been proposed. For example, ESMFold [85], developed by Meta AI, used the information and representations learned by a PLM called ESM-2 to perform end-to-end 3D structure prediction using only a single sequence as input. ESMFold demonstrated comparable accuracy to AlphaFold2 and RoseTTAFold for sequences exhibiting low perplexity and thorough comprehension by PLM. Notably, ESMFold’s inference speed was ten times faster than that of AlphaFold2, thereby facilitating efficient exploration of the structural landscape of proteins within practical time frames. OmegaFold [121] predicted the high-resolution protein structure from a single primary sequence alone, using a combination of a PLM and a geometry-inspired transformer model, trained on protein structures. OmegaFold requires only a single amino acid sequence for protein structure prediction and does not rely on MSAs or known structures as templates. Similar to ESMFold, OmegaFold can also scale roughly ten times faster than MSA-based methods, such as AlphaFold2 and RoseTTAFold. HelixFold-Single [119] was an end-to-end MSA-free protein structure prediction pipeline that combined a large-scale PLM with the superior geometric learning capability of AlphaFold2. HelixFold-Single first pre-trained a large-scale PLM with thousands of millions of primary structures, utilizing the self-supervised learning paradigm, and then obtained an end-to-end differentiable model to predict 3D structures by combining the pre-trained PLM and the essential components of AlphaFold2. EMBER3D [122] predicted 3D structure directly from single sequences by computing both 2D (distance maps) and 3D structure (backbone coordinates) from sequences alone, based on embeddings from the pre-trained PLM called ProtT5. EMBER3D exhibited a speed that was orders of magnitude faster than its counterparts, enabling the prediction of average-length structures in mere milliseconds, even on consumer-grade machines.

The benchmark results in Table 1 and Table 2 indicate that PLM-based protein structure prediction methods are generally worse than MSA-based methods, although PLM-based methods run very fast. Due to the large scalability of PLM-based methods, they have broad application prospects, and still require further improvements in terms of accuracy.

### 2.7. Multi-Domain Protein Structure Prediction

Since the advent of AlphaFold2 in the recent CASP14, great progress has been made in protein structure prediction. However, AlphaFold2 and most of the subsequent state-of-the-art methods have mainly focused on the modeling of single-domain proteins, which are the minimum folding units of proteins that fold and function independently. Nonetheless, it is worth noting that several of the CASP14 targets, especially large multi-domain targets, were not predicted with high accuracy, suggesting that further improvements are needed for multi-domain prediction [123]. As shown in Table 1 and Table 2, AlphaFold2 had an average TM-score of 0.8871 on domain-level assessments, but only 0.8514 when considering multi-domain targets. This is because the full-length-level assessments account for multi-domain targets, where AlphaFold2 still needs to be improved. In fact, more than two-thirds of prokaryotic proteins and four-fifths of eukaryotic proteins contain two or more domains [124]. Therefore, determining the full-length structures of multi-domain proteins is highly required.

A common approach to multi-domain protein structure modeling is to split the query sequence into domains and generate models for each individual domain separately. The individual domain models are subsequently assembled into full-length models, usually under the guidance of other homologous multi-domain proteins from the PDB. Such domain assembling methods can be divided into the following two categories: linker-based domain assembly and inter-domain rigid body docking. Linker-based methods, such as Rosetta [125] and AIDA [126], primarily focus on the construction of linker models by exploring the conformational space, with domain orientations loosely constrained by physical potential from generic hydrophobic interactions. Docking-based methods, such as DEMO [127,128] and SADA [129], assemble the single domain structure via rigid body docking, which is essentially a template-based method that guides domain assembly by detecting available templates.

Furthermore, some fully automated pipelines [130] for multi-domain protein structure prediction from sequences alone have been developed based on this idea. For example, I-TASSER-MTD first predicted domain boundaries from sequences by FUpred [131] and ThreaDom [132]. Then, single-domain structural models were folded by the original version of D-I-TASSER [108] guided by deep-learning spatial restraints [100,101]. Finally, DEMO [127,128] was used to perform multi-domain structure assembly.

Note that the performance of common protein structure prediction methods relies, to some extent, on the quality of the MSA or the homologous template [66]. However, homologs available in the PDB may be fewer for multi-domain proteins, which may further affect the performance of multi-domain protein structure prediction. Thus, some threading-based methods, such as LOMETS3 [77], have been developed to increase template recognition and alignment accuracy for multi-domain proteins. LOMETS3 performed three steps of domain boundary prediction, domain-level template identification, and full-length template/model assembly, which can help better detect distant homologous templates for multi-domain proteins [77]. Furthermore, the DeepMSA2 [133] algorithm has been proposed to generate deeper MSAs, facilitating the improvement in MSA quality for multi-domain protein structure prediction.

Aside from the challenges presented by shallow MSAs, another significant limitation in multi-domain protein structure prediction is accurately modeling the orientation between different domains. Some efforts have been made to improve the inter-domain orientation problem in multi-domain protein structure prediction. For example, DeepAssembly [134] used a population-based evolutionary algorithm to assemble multi-domain proteins, leveraging inter-domain interactions inferred from a developed deep learning network. E2EDA [135] was an end-to-end domain assembly method based on deep learning. It first predicted inter-domain rigid motion using an attention-based deep learning model. Subsequently, these predicted rigid motions were translated into inter-domain spatial transformations to allow for the direct assembly of full-chain models. The final stage involved selecting the best model from multiple assembled models, guided by a specific scoring strategy.

**Figure 6:**
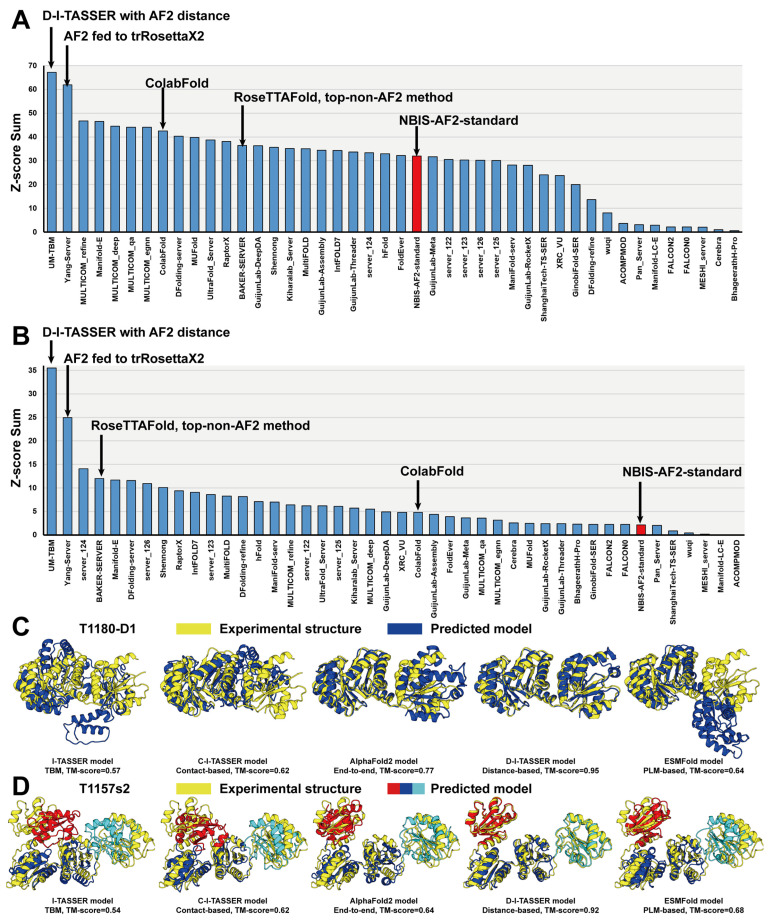
Protein structure prediction results in CASP15. (**A**,**B**) Sums of Z-scores for the top 44 registered server groups in the (**A**) “Regular Modeling” and (**B**) “Inter-domain Modeling” Sections in CASP15. The public version 2.2.0 of the AlphaFold2 server (registered as “NBIS-AF2-standard”) is marked in red. (**C**,**D**) The modeling performance of I-TASSER (a template-based modeling (TBM) method), C-I-TASSER (a contact-based method), D-I-TASSER (a distance-based method), AlphaFold2 (an end-to-end method), and ESMFold (a protein language model (PLM)-based method) on representative examples of (**C**) CASP15 single-domain target T1180-D1 and (**D**) CASP15 multi-domain target T1157s2. The single-domain predicted models are depicted in blue, the multi-domain predicted models are marked by red, blue, and cyan to distinguish different domains, and the superposed experimental structures are represented by yellow.

Furthermore, the latest version of the D-I-TASSER pipeline has been developed by integrating all aforementioned strategies to improve multi-domain protein structure predictions. D-I-TASSER first generated MSAs by DeepMSA2 [133], which were then used for template identification by LOMETS3 [77] and spatial restraint prediction by AlphaFold2, AttentionPotential [133], and DeepPotential [136], on both the full-length level and the domain level, with the aid of a multi-domain handling module that incorporated FUpred [131], ThreaDom [132], and DEMO2 [127]. Unlike I-TASSER-MTD, which attempted to assemble domain-level models into the full-length model, D-I-TASSER directly predicted the full-length atomic model from both full-length-level inputs and domain-level assembled inputs, that is, the templates and spatial restraints, through the Replica Exchange Monte Carlo (REMC) folding system [91,92,93]. In this way, the inter-domain orientation information contained in full-length-level inputs can be used to construct the final model. D-I-TASSER (named as “UB-TBM”) participated in the CASP15 “Inter-domain Modeling” Section, which corresponds to multi-domain structure prediction. D-I-TASSER outperformed all other groups in terms of the Z-score sum, calculated by the CASP Assessors (Figure 6). In particular, the Z-score sum of D-I-TASSER (35.53) was 42.3% higher than that of the second-best performing group (24.96) (see https://predictioncenter.org/casp15/zscores_interdomain.cgi, accessed on 10 December 2023).

### 2.8. CASP and Most Recent CASP Results

The Critical Assessment of Protein Structure Prediction (CASP) was established in 1994, by Professor John Moult and others from the University of Maryland, and has taken place every other year since then [137]. Its purpose is to provide an objective evaluation of protein structure prediction technologies within the field of protein structure prediction. Employing a rigorous double-blind prediction mechanism, it is viewed as the gold standard for assessing protein structure prediction techniques and is regarded in the industry as the “Olympics of protein structure prediction”.

In order to fairly evaluate protein structure prediction methods, CASP assessors have incorporated and designed multiple measures. Two widely used evaluation measures by CASP are the TM-score and the global distance test score (GDT score). The TM-score between the model and the experimental structure is usually used to assess the global quality of a structural model [138]. The TM-score ranges between 0 and 1, with TM-scores > 0.5 indicating that the structure models have the same fold defined in SCOP/CATH [117]. The GDT score is calculated by GDT = (GDT_P1 + GDT_P2 + GDT_P4 + GDT_P8)/4, where GDT_Pn indicates the percent of residues under the distance cut-off ≤ n Å [139]. The GDT score primarily focuses on assessing the backbone modeling quality of a protein. With the substantial enhancement in prediction accuracy witnessed since the advent of AlphaFold2 in CASP14, more and more measures for assessing side-chain modeling quality have been introduced. For instance, SC_error is a measure used for assessing side-chain modeling quality, while MolProbity is a comprehensive scoring function used for assessing the non-physical area of the model (i.e., atom clash, rotamer outlier, favored Ramachandran, etc.).

According to the rules of CASP, all participating methods are categorized into the following two groups: server-based and human-based. Participants in the server-based group have a limited window of 72 h for structure prediction, while those in the human-based group are allotted 3 weeks, allowing for manual intervention. This signifies that the server-based group relies solely on computer predictions; hence, the competitive difficulty in this category is often higher than in the human-based groups.

Starting from CASP7, the proteins modeled during CASP have been classified as TBM, TBM-easy, TBM-hard, FM/TBM, or FM, depending on the availability and quality of PDB templates for each target, where TBM-easy targets have readily identifiable, high-quality templates, and FM targets typically lack homologous templates in the PDB. For the purpose of analyses, TBM, TBM-easy, and TBM-hard are often regarded as TBM targets, and FM/TBM and FM are treated as FM targets.

Starting from CASP12, protein complex prediction has been included in CASP as an independent assessment category, called the protein assembly category. Protein complex modeling is distinguished from the classical protein–protein docking, where two protein subunits, named the ligand and the receptor, are in contact through a single interface. In the CASP protein assembly assessment, predictions of full-length protein complexes involve predictions of both individual protein–protein interfaces and overall complex topology.

Starting from CASP13, deep learning techniques have achieved significant breakthroughs, markedly enhancing the accuracy of protein tertiary structure prediction.

In CASP13, the adoption of distance map prediction began to play a pivotal role in guiding protein structure prediction. Notable examples include RaptorX-Contact [22], DMPfold [105], and AlphaFold [106], which employed deep Residual Networks (ResNets) from contact prediction to distance prediction, significantly boosting predictive modeling performance. In particular, AlphaFold, developed by Google DeepMind, was ranked as the top method in tertiary structure modeling among all groups in CASP13. However, the majority of other groups continued to rely on contact prediction information for guiding protein structure prediction. Due to the remarkable accuracy of deep learning-based contact map predictions, even contact-based protein structure prediction methods also achieved excellent performance. For instance, C-I-TASSER and C-QUARK were ranked as the top two automated servers during CASP13 [23].

The effectiveness of distance prediction, as demonstrated in CASP13, has led to its widespread applications in various structure prediction methodologies. A promising example is trRosetta [25,107], which employed a deep residual neural network to predict both pairwise residue distances and inter-residue orientations for guiding protein structure prediction. Following the inspiration from trRosetta, numerous groups in CASP14 incorporated orientation and distance constraints predicted by deep residual neural networks into their protein structure prediction processes. Among these methods, D-I-TASSER [108] and D-QUARK [108] were two top CASP14 servers from Yang Zhang’s group. D-I-TASSER, in particular, leveraged deep learning-based hydrogen bond network prediction to guide protein structure prediction, significantly improving modeling accuracy for CASP14 targets, especially those lacking homologous templates [108]. More importantly, AlphaFold2 represented a groundbreaking shift by employing an end-to-end deep learning approach to protein structure prediction, and facilitated the rise of predictive performance to unprecedented levels, regularly competitive with experimental structures in CASP14.

In CASP15, following the release of the AlphaFold2 codes, most groups adopted the AlphaFold2 framework for their structure predictions, resulting in outstanding performance across the board. Figure 6A,B list the sums of Z-scores, calculated by the CASP Assessors, for the top 44 CASP15 server groups that participated in the CASP15 “Regular Modeling” (https://predictioncenter.org/casp15/zscores_final.cgi?formula=assessors&gr_type=server_only, accessed on 10 December 2023) and “Inter-domain Modeling” (https://predictioncenter.org/casp15/zscores_interdomain.cgi, accessed on 10 December 2023) Sections, which correspond to single- and multi-domain structures, respectively. Here, we only show the results from server groups because the human group results may incorporate experience and expertise, which may be unfair for evaluating different protein structure prediction methods. In CASP15, due to the release of the AlphaFold2 standalone package, most of the participant methods were AlphaFold2-based methods. In particular, the top five performing methods were all based on AlphaFold2, with their own modifications, such as incorporating AlphaFold2 with other simulation pipelines, using diverse MSAs, and fine-tuning AF2 refinements; thus, they acquired much better performance than the default AlphaFold2 (registered as the “NBIS-AF2-standard” group). The top non-AlphaFold2 method was based on RoseTTAFold2 (registered as the “BAKER” group), which had good predictive performance on multi-domain proteins. In Figure 6C,D, we used representative examples of a single-domain target, T1180-D1, and a multi-domain target, T1157s2, from CASP15 to highlight the modeling performance of different types of methods, including a template-based modeling (TBM) method, I-TASSER, a contact-based method, C-I-TASSER, a distance-based method, D-I-TASSER, an end-to-end method, AlphaFold2, and a protein language model (PLM)-based method, ESMFold. The TBM method exhibited the worst performance, with TM-scores of 0.57 and 0.54 for the single-domain and the multi-domain targets, respectively. The contact-based method also showed limited accuracy for both targets. AlphaFold2, the recently developed end-to-end method, demonstrated improved performance on the single-domain target (TM-score = 0.77) but slightly reduced efficacy on the multi-domain target (TM-score = 0.64), highlighting the inherent challenges in multi-domain protein structure prediction. Notably, the latest version of D-I-TASSER achieved remarkable predictive accuracy for both single-domain and multi-domain targets by carefully integrating the AlphaFold2 pipeline with a multi-domain handling module. On the other hand, despite its rapid execution, the PLM-based method exhibited suboptimal performance, particularly on the single-domain target.

In particular, CASP15 introduced a new category, ligand prediction, where participants were provided with both protein (or RNA) and ligand data to generate 3D structural models for the corresponding protein/RNA–ligand complexes [140]. All leading groups in this category adopted similar methodologies, which started from a search in the PDB for similar ligands and binding pockets. Following this, the identified PDB binding pockets were superimposed onto the AlphaFold2 structures of the target proteins. This superposition facilitated the generation of an initial pose for the ligand. To further refine and evaluate these alignments, various conventional methods and machine learning techniques were employed.

For example, the CoDock approach [141] combined template-based modeling with a convolutional neural network (CNN)-based scoring function to predict ligand binding. The Zou group [142] adopted a similar strategy, integrating the physicochemical molecular docking method AutoDock Vina [143] with the ligand similarity methodology SHAFTS [144]. In the Alchemy_LIG team [145] protein structures were constructed using AlphaFold2, and ligands were docked utilizing the AutoDock Vina docking method and a machine learning model trained to detect native binding modes. The ClusPro group [146] employed AlphaFold2 for constructing monomer protein structures and created multimeric assemblies via a template-based docking algorithm, ClusPro LigTBM [146], for general ligand placement, alongside the Glide program [147], for direct docking in cases when no templates were found.

While docking approaches utilizing templates from the PDB demonstrated superior performance, it is important to recognize that the excellent performance of these template-based methods was not uniformly observed across all CASP15 targets [140]. Furthermore, it is noteworthy that state-of-the-art deep learning techniques have yet to be extensively employed in the realm of protein–ligand structure predictions, representing a significant and promising avenue for future research.

### 2.9. AlphaFold Protein Structure Database (AlphaFold DB)

The AlphaFold Protein Structure Database (AlphaFold DB, https://alphafold.ebi.ac.uk, accessed on 10 December 2023), created in partnership between DeepMind and the EMBL-European Bioinformatics Institute (EMBL-EBI), is a freely accessible database of high-accuracy protein structure predictions by the scientific community [148]. Powered by AlphaFold2 of Google DeepMind, AlphaFold DB provides highly accurate protein structure predictions, competitive with experimental structures. The latest AlphaFold DB release contains over 200 million entries, providing broad coverage of UniProt [149], which is the standard repository of protein sequences and annotations. AlphaFold DB provides individual downloads for the human proteome and for the proteomes of 47 other key organisms important in research and global health. AlphaFold DB also provides a download for the manually curated subset of UniProt. The prediction results of AlphaFold DB can be accessed through several mechanisms, as follows: (i) bulk downloads (up to 23 TB) via FTP; (ii) programmatic access via an application programming interface (API); and (iii) download and interactive visualization of individual predictions on protein-specific web pages keyed on UniProt accessions.

The AlphaFold DB’s release of a multitude of novel protein structures has provided bioinformaticians across the globe with a rich repository of data. Developers specializing in protein structure analysis tools are leveraging this influx of accurate models, leading to numerous significant breakthroughs in protein-related fields.

For example, the AlphaFold DB, through its accurate prediction of protein structures, offers a robust foundation for understanding how different ligands might interact with various proteins, which is pivotal in identifying potential drug targets, aiding in the design of novel pharmaceuticals, and contributing to a broader understanding of biological functions. In this context, several methods have been developed. AlphaFill, for instance, was developed to enrich the models in the AlphaFold DB by “transplanting” ligands, co-factors, and ions, based on sequence and structure similarity [150]. Similarly, Wehrspan et al. investigated the binding sites for iron–sulfur (Fe-S) clusters and zinc (Zn) ions within predicted structures in AlphaFold DB [151]. With the utilization of the AlphaFold DB, PrankWeb3 was able to predict protein–ligand binding sites in situations where no experimental structure is available [152].

Another recent application of AlphaFold DB was related to post-translation modifications (PTMs) [153], where structural insights obtained from AlphaFold DB were systematically integrated with proteomics data, particularly large-scale PTM information, aiming to illuminate the functional significance of PTMs.

While the AlphaFold DB has significantly expanded the application and scalability of tools and algorithms for protein-related analyses, effectively analyzing more than a couple of hundred thousand protein structures or models poses a challenge. There is a pressing need to develop novel approaches capable of managing the unanticipated and rapid growth of available models. Notably, state-of-the-art tools such as FoldSeek [154] and 3D-AF-Surfer [155] have already been developed, aiding researchers in searching through extensive repositories of protein structures to identify hits with structural similarity to a provided input structure. Leveraging high-throughput structural similarity searches facilitates classification problems, such as assigning structural CATH domains to AlphaFold models [156].

However, many limitations and challenges still remain for AlphaFold DB, such as predicting multi-domain protein structures, and predicting structures for very large proteins (longer than 5000 residues) [157].

## 3. Discussion and Perspective

Since Anfisen first demonstrated that the information encoded in a protein sequence determines its structure [1], the prediction of protein structures starting from amino acid sequences has remained a challenging problem in structural biology. A number of methods have been proposed to address the problem of protein structure prediction.

The traditional approaches for solving the protein structure prediction problem involve template-based modeling (TBM) and template-free modeling (FM) methods. The TBM approaches demonstrate high efficacy when homologous templates are easily identifiable. However, their accuracy significantly decreases in cases where only distantly related templates are available for a target (see Table 3). On the other hand, FM methods are generally limited to folding smaller, non-beta proteins because of the computational complexities inherent in their energy functions and conformational sampling techniques.

Recent breakthroughs in deep learning-based restraint prediction and end-to-end folding have significantly revolutionized the field of protein structure prediction. These developments have markedly improved prediction accuracy and the ability to fold proteins that lack corresponding homologous templates in the PDB. In particular, AlphaFold2 and subsequent methodologies have largely tackled the challenge of protein structure prediction at the domain level through the implementation of end-to-end learning and attention-based networks. However, the predictive accuracy of these AlphaFold2-based methods is significantly dependent on the quality of multiple sequence alignments (MSAs). To bypass the over-reliance on MSAs, protein language model (PLM)-based methods have been developed as alternatives to MSAs for acquiring co-evolutionary information, thus enabling MSA-free predictions. Although these PLM-based approaches are notably rapid, due to the absence of MSA construction, their performance still requires further improvements.

It is crucial to note that neither end-to-end methods nor PLM-based methods can predict multi-domain proteins with high accuracy. Consequently, many methods have been designed for multi-domain protein structure predictions in particular. Nevertheless, substantial challenges persist, particularly in the construction of high-quality MSAs and the accurate modeling of orientations between disparate domains. While some advancements have been made to solve these limitations, there remains a need for further improvements in multi-domain protein prediction, as demonstrated by the generally reduced performance in the “Inter-domain Modeling” Section of CASP15 (Figure 6B).

While the majority of structure prediction methods are based on static structures, it is crucial to recognize that proteins often exist in multiple conformational states, intricately linked to their distinctive functional roles. Notably, the understanding of protein conformational states and folding pathways is critically important in drug development. Furthermore, conformational changes are a key concern in protein–ligand prediction. The principal challenge in this area comes from the limited availability of data on protein motion and evolutionary information. With the increasing number of experimental data, it is expected that more and more methods will be developed to address these challenges [158,159,160]. Particularly, AlphaFold DB, with its remarkable accuracy in predicting protein structures, has facilitated improvements in this field. For instance, AlphaFold2 successfully demonstrated its ability to identify alternative states of known metamorphic proteins with high confidence by clustering a MSA based on sequence similarity, indicating a significant leap forward in understanding protein dynamics [159]. In addition, a recent study introduced a methodology that utilized AlphaFold2 to sample alternative conformations of topologically diverse transporters and G-protein-coupled receptors, which were not included in the AlphaFold2 training dataset [160].

Due to the high accuracy of recent protein structure prediction methods, these methods can effectively help biologists conduct protein structure and function analyses, for example, using protein structure prediction to assist cryo-electron microscopy electron density maps to resolve atomic-level experimental structures [161,162] and analyzing the structural and functional differences of specific proteins from different species through protein structure prediction methods [163]. In particular, during the novel coronavirus pneumonia outbreak at the end of 2019, no protein structures of the virus were initially analyzed. Given the critical role of the viral proteome as a functional carrier, understanding its structure was important for analyzing the mechanism of viral host invasion. Consequently, several research groups have predicted the full proteome of the SARS-CoV-2 virus, as well as the spike protein of the mutant virus [164,165], and made these predictions freely available in databases for biological researchers.

As protein monomer structure predictions have achieved high accuracy, more and more attention has shifted toward protein complex structure predictions and RNA-related structure predictions. For example, advanced protein structure prediction approaches have been extended to protein complex structure prediction [133,166]. Since most proteins cooperate with their protein interaction partners to form a complex for performing their biological functions in biological processes within a living cell, various experimental methods have been proposed to detect protein complexes, such as AlphaFold-Multimer [166] and DMFold-Multimer [133]. A primary challenge in complex prediction lies in the substantial computational resources necessary for the prediction of large, multi-chain proteins. Furthermore, acquiring high-quality MSAs for complexes is also a particularly challenging task.

Another extension of protein structure prediction involves RNA structure prediction [167,168,169] and RNA–protein complex structure prediction [170], where representative methods include AIchemy_RNA2 [167], DRfold [168], trRosettaRNA [169], and RoseTTAFoldNA [170]. Despite the increasing accumulation of experimental structural data for RNA, the field of RNA or RNA–protein structure prediction is still challenged by the limited availability of RNA sequence and structure databases, as well as the complexities in extracting conservation information from RNA sequences. As demonstrated by CASP 15, deep learning-based RNA structure predictors did not surpass the performance of traditional energy function-based methods because the performance of deep learning-based methods heavily relies on the number of training data available. The accuracy of RNA structure predictions, whether obtained through traditional or deep learning methodologies, is far from satisfactory.

Although AlphaFold2 and many state-of-the-art methods constitute a significant advancement in “solving” the problem of protein structure prediction from sequences, they are not the final answer. There are still challenges met in searching for high-quality MSAs, improving the side-chain modeling quality [171], and so on. Furthermore, challenges in protein complex structure predictions, RNA-related structure predictions, and protein–ligand structure predictions have received growing attention. The rapid progress observed in recent years brings hope that the problems and challenges associated with protein structure prediction could ultimately be solved by leveraging deep learning techniques in the future.

## Figures and Tables

**Table 1:** Comparison of domain-level modeling results by AlphaFold2-based methods and protein language model (PLM)-based methods for different domain types on the 91 CASP14 domains. The original CASP “TBM-easy” and “TBM-hard” domains are categorized as “TBM” domains, while the “FM/TBM” and “FM” domains are categorized as “FM” domains in this analysis. Here, AlphaFold2-Single is the default AlphaFold2 pipeline, with the only query sequence as the input MSA. *p*-values were calculated between TM-scores by AlphaFold2 and others using paired one-sided Student’s *t*-tests. #{TM > 0.5} is the number of targets with a TM-score > 0.5.

Method	Method Type	Domain Type	TM-Score	*p*-Value	#{TM > 0.5}
AlphaFold2	AlphaFold2-based	All	0.8871	-	88
		TBM	0.9325	-	54
		FM	0.8207	-	34
ColabFold		All	0.8846	3.29 × 10^−2^	88
		TBM	0.9255	6.65 × 10^−3^	54
		FM	0.8250	4.25 × 10^−1^	34
OpenFold		All	0.8692	3.17 × 10^−2^	85
		TBM	0.9199	1.57 × 10^−1^	53
		FM	0.7952	5.24 × 10^−2^	32
Uni-Fold		All	0.8930	9.93 × 10^−1^	88
		TBM	0.9387	9.76 × 10^−1^	54
		FM	0.8262	9.26 × 10^−1^	34
AlphaFold2-Single	PLM-based	All	0.5165	4.06 × 10^−16^	40
		TBM	0.6609	5.15 × 10^−10^	37
		FM	0.3057	2.18 × 10^−11^	3
ESMFold		All	0.7206	4.64 × 10^−14^	66
		TBM	0.8481	1.89 × 10^−7^	50
		FM	0.5346	1.02 × 10^−10^	16
OmegaFold		All	0.6920	4.60 × 10^−9^	64
		TBM	0.7944	5.42 × 10^−6^	46
		FM	0.5426	2.18 × 10^−5^	18

**Table 2:** Comparison of full-length-level modeling results by AlphaFold2-based methods and protein language model (PLM)-based methods on the 65 CASP14 full-length targets. Here, AlphaFold2-Single is the default AlphaFold2 pipeline, with the only query sequence as the input MSA. *p*-values were calculated between TM-scores by AlphaFold2 and others using paired one-sided Student’s *t*-tests. #{TM > 0.5} is the number of targets with a TM-score > 0.5.

Method	Method Type	TM-Score	*p*-Value	#{TM > 0.5}
AlphaFold2	AlphaFold2-based	0.8514	-	60
ColabFold		0.8461	2.37 × 10^−1^	61
OpenFold		0.8375	1.46 × 10^−1^	59
Uni-Fold		0.8561	9.82 × 10^−1^	61
AlphaFold2-Single	PLM-based	0.5164	6.88 × 10^−12^	30
ESMFold		0.6676	1.80 × 10^−10^	41
OmegaFold		0.6728	4.42 × 10^−6^	43

**Table 3:** The advantages and limitations of each type of methods.

Method	Advantages	Limitations
Template-based modeling (TBM)	The methods can achieve high accuracy and adeptly reflect evolutionary relationships when reliable templates are identifiable.	The accuracy of TBM significantly decreases when the available templates are only distantly related to the target protein.
Template-free modeling (FM)	The methods are not limited to the availability of templates and, thus, can be applied to any protein.	The statistical and knowledge-based energy potentials used in FM methods may lead to suboptimal performance if they are inaccurate. Also, these energy potentials contain little residue–residue interaction information.
Contact/distance-based methods	The energy potentials derived from deep learning-based restraints (contacts or distances) contain high-quality residue–residue interaction information.	The deep learning-based restraints (contacts or distances) and the final structural models are optimized separately, which may be difficult for improving overall accuracy. Additionally, the requirement for MSA inputs poses a challenge for distance-based methods, especially in cases in which high-quality MSAs are difficult to obtain.
End-to-end methods	The deep learning-based restraints (contacts or distances) and the final structural models are optimized together, resulting in their high accuracy in single-domain proteins.	Such methods have shown limitations in accurately predicting the structures of multi-domain proteins, especially for proteins with few known homologs.
Protein language model (PLM)-based methods	These methods have high scalability and computational efficiency, since they do not rely on MSA inputs. Also, their performance is relatively better for orphan proteins.	PLM-based methods currently suffer from relatively low accuracy in structure prediction.
Multi-domain protein structure prediction methods	These methods are well-designed for multi-domain proteins, with high performance to balance the modeling quality of inter-domain and intra-domain interactions.	These methods face challenges in carefully balancing MSAs for both separate domains and full-length proteins, accurately modeling the orientations between disparate domains, and predicting the accurate domain boundaries.

## Data Availability

No new data were created or analyzed in this study. Data sharing is not applicable to this article.

## Supplementary Materials

The following supporting information can be downloaded at: https://www.mdpi.com/article/10.3390/molecules29040832/s1, Figure S1: An illustration of co-evolutionary information contained in multiple sequence alignments and the corresponding relationships with residue–residue contact prediction; Table S1: Tools for template-based protein structure prediction; Table S2: Tools for template-free (free modeling) protein structure prediction; Table S3: Tools for contact-based protein structure prediction; Table S4: Tools for distance-based protein structure prediction; Table S5: Tools for end-to-end protein structure prediction; Table S6: Tools for protein language model-based protein structure prediction; Table S7: Tools for multi-domain protein structure prediction; Table S8: The monomer protein dataset from CASP14 used in our benchmark tests. Refs. [172,173,174,175,176,177,178,179,180,181,182,183,184,185,186,187,188,189] are cited within the Supplementary Materials.

## Abbreviations

3D, three-dimensional; AI, artificial intelligence; AlphaFold DB, AlphaFold Protein Structure Database; API, application programming interface; CASP, Critical Assessment of Protein Structure Prediction; CNN, convolutional neural network; DCA, direct coupling analysis; EMBL-EBI, EMBL-European Bioinformatics Institute; Fe-S, iron–sulfur; FM, template-free modeling; GDT, global distance test; HMM, hidden Markov model; MD, molecular dynamics; MSA, multiple sequence alignment; PDB, Protein Data Bank; PLM, protein language model; PSSM, position-specific score matrix; PTM, post-translation modification; REMC, Replica Exchange Monte Carlo; ResNet, deep residual network; TBM, template-based modeling; Zn, zinc.
