# Supplementary material for: Recent Progress of Protein Tertiary Structure Prediction
Source: Molecules. 2024 Feb 13;29(4):832. doi: 10.3390/molecules29040832 (PMC10893003; doi:10.3390/molecules29040832)
Supplement: Supplementary file 1 [file molecules-29-00832-s001.zip › molecules-2826068-supplementary.pdf]

Supplementary Information for

Recent progress of protein tertiary structure prediction

Qiqige Wuyun, Yihan Chen, Yifeng Shen, Yang Cao, Gang Hu, Wei Cui, Jianzhao Gao, Wei Zheng

Table of Content

Supplementary Figures

Fig. S1. An illustration of co-evolutionary information contained in multiple sequence alignments and the corresponding relationship with residue-residue contact prediction.

Supplementary Tables

Table S1. Tools for template-based protein structure prediction.
Table S2. Tools for template free (free modeling) protein structure prediction.
Table S3. Tools for contact-based protein structure prediction.
Table S4. Tools for distance-based protein structure prediction.
Table S5. Tools for end-to-end protein structure prediction.
Table S6. Tools for protein language model-based protein structure prediction.
Table S7. Tools for multi-domain protein structure prediction.
Table S8. The monomer protein dataset from CASP14 used in our benchmark tests.

Supplementary Figures

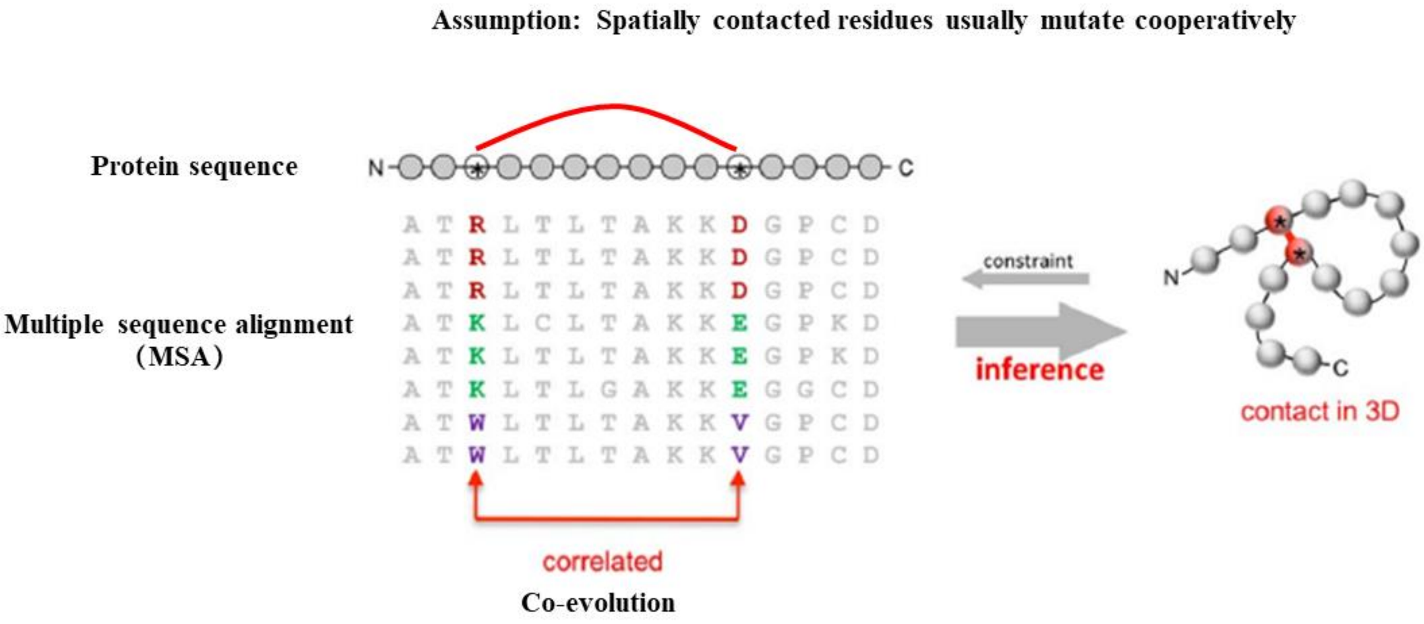

Figure S1. An illustration of co-evolutionary information contained in multiple sequence alignments and the corresponding relationship with residue-residue contact prediction.

Supplementary Tables

Table S1. Tools for template-based protein structure prediction.

Methods	URLs	Reference
CEthreader	https://zhanglab.dcmb.med.umich.edu/CEthreader/	[1]
DEDAL	https://github.com/google-research/google-research/tree/master/dedal	[2]
DisCovER	https://github.com/Bhattacharya-Lab/DisCovER	[3]
EBA	https://git.scicore.unibas.ch/schwede/EBA<br>https://git.scicore.unibas.ch/schwede/eba_benchmark	[4]
EigenTHREADER	https://github.com/psipred/eigenthreader	[5]
FFAS-3D	https://ffas.godziklab.org/ffas-cgi/cgi/ffas.pl	[6]
HHpred	https://github.com/soedinglab/hh-suite/tree/master/scripts/hhpred	[7]
HHsearch	https://github.com/soedinglab/hh-suite	[8]
I-TASSER	https://zhanglab.dcmb.med.umich.edu/I-TASSER/	[9-11]
LOMETS	https://zhanglab.dcmb.med.umich.edu/LOMETS/	[12-14]
map_align	https://github.com/sokrypton/map_align<br>https://github.com/gjoni/map_align	[15]
MODELLER	https://salilab.org/modeller/	[16]
MRFalign	http://raptorx6.uchicago.edu	[17]
MULTICOM	https://multicom-toolbox.mu.hekademeia.org	[18, 19]
MUSTER	https://zhanglab.dcmb.med.umich.edu/MUSTER/	[20]
Phyre2	http://www.sbg.bio.ic.ac.uk/phyre2/	[21]
pLM-BLAST	https://toolkit.tuebingen.mpg.de/tools/plmblast<br>https://github.com/labstructbioinf/pLM-BLAST	[22]
RaptorX	https://github.com/j3xugit/RaptorX-3DModeling<br>http://raptorx6.uchicago.edu	[23, 24]

RosettaCM	https://www.rosettacommons.org/software/license-and-download	[25]
SAdLSA	https://sites.gatech.edu/cssb/sadlsa/	[26]
SparksX	https://sparks-lab.org/server/sparks-x/	[27]
ThreaderAI	https://github.com/ShenLab/ThreaderAI	[28]

Table S2. Tools for template free (free modeling) protein structure prediction.

Methods	URLs	Reference
FragFold	https://github.com/psipred/fragfold	[29]
QUARK	https://zhanglab.dcmb.med.umich.edu/QUARK/	[30]
Rosetta	https://robetta.bakerlab.org<br>https://www.rosettacommons.org/software/license-and-download	[31, 32]

Table S3. Tools for contact-based protein structure prediction.

Methods	URLs	Reference
CEthreader	https://zhanglab.dcmb.med.umich.edu/CEthreader/	[1]
C-I-TASSER	https://zhanglab.dcmb.med.umich.edu/C-I-TASSER/	[33]
CONFOLD2	https://github.com/multicom-toolbox/CONFOLD2/	[34]
C-QUARK	https://seq2fun.dcmb.med.umich.edu/C-QUARK/	[35]
EigenTHREADER	https://github.com/psipred/eigenthreader	[5]
LOMETS2	https://zhanglab.dcmb.med.umich.edu/LOMETS/	[12-14]
map_align	https://github.com/sokrypton/map_align<br>https://github.com/gjoni/map_align	[15]
RaptorX-Contact	https://github.com/j3xugit/RaptorX-Contact	[36]

Table S4. Tools for distance-based protein structure prediction.

Methods	URLs	Reference
DeepFold	https://zhanggroup.org/DeepFold/	[37]
DeepPotential	https://zhanggroup.org/DeepPotential/	[38]
DeepThreader	http://raptorx6.uchicago.edu	[39]
DisCovER	https://github.com/Bhattacharya-Lab/DisCovER	[3]
D-I-TASSER	https://zhanggroup.org/D-I-TASSER/	[40, 41]
DMPfold	http://bioinf.cs.ucl.ac.uk/psipred/<br>https://github.com/psipred/DMPfold	[42]
D-QUARK	https://zhanggroup.org/D-QUARK/	[41]
tFold	https://drug.ai.tencent.com/console/en/tfold	[43]
trRosetta	https://yanglab.qd.sdu.edu.cn/trRosetta/<br>https://github.com/gjoni/trRosetta<br>https://github.com/RosettaCommons/trRosetta2	[44, 45]

Table S5. Tools for end-to-end protein structure prediction.

**Methods**	**URLs**	**Reference**
AlphaFold2	https://github.com/deepmind/<br>https://github.com/google-deepmind/alphafold	[46]
ColabFold	https://github.com/sokrypton/ColabFold	[47]
FastFold	https://github.com/hpcaitech/FastFold	[48]
HelixFold	https://github.com/PaddlePaddle/PaddleHelix/tree/dev/apps/protein_folding/helixfold	[49]
MEGA-Fold	https://gitee.com/mindspore/mindscience/tree/master/MindSPONGE/applications/MEGAProtein	[50]
OpenFold	https://github.com/aqlaboratory/openfold	[51]
RoseTTAFold	https://robetta.bakerlab.org<br>https://github.com/RosettaCommons/RoseTTAFold	[52]
Uni-Fold	https://github.com/dptech-corp/Uni-Fold	[53]

Table S6. Tools for protein language model-based protein structure prediction.

**Methods**	**URLs**	**Reference**
EMBER3D	https://github.com/kWeissenow/EMBER3D	[54]
ESM-Fold	https://github.com/facebookresearch/esm	[55]
HelixFold-Single	https://github.com/PaddlePaddle/PaddleHelix/tree/dev/apps/protein_folding/helixfold-single	[56]
IgFold	https://github.com/Graylab/IgFold	[57]
OmegaFold	https://github.com/HeliXonProtein/OmegaFold	[58]
RaptorX-Single	https://github.com/AndersJing/RaptorX-Single	[59]
trRosettaX-Single	https://yanglab.qd.sdu.edu.cn/trRosetta/benchmark_single	[60]

Table S7. Tools for multi-domain protein structure prediction.

**Methods**	**URLs**	**Reference**
AIDA	http://ffas.burnham.org/AIDA/	[61]
DeepAssembly	http://zhanglab-bioinf.com/DeepAssembly/	[62]
DEMO	https://zhanggroup.org/DEMO/	[63, 64]
D-I-TASSER	https://zhanggroup.org/D-I-TASSER/	[40, 41]
E2EDA	http://zhanglab-bioinf.com/E2EDA/	[65]
I-TASSER-MTD	https://zhanggroup.org/I-TASSER-MTD/	[66]
LOMETS3	https://zhanglab.dcmb.med.umich.edu/LOMETS/	[12-14]
SADA	http://zhanglab-bioinf.com/SADA/	[67]

Table S8. The CASP14 benchmark dataset, including 65 full-length proteins and the corresponding 91 domains. The 91 domains can be divided as 54 TBM domains and 37 FM domains. The original CASP 'TBM-easy' and 'TBM-hard' domains are categorized as 'TBM' domains, while 'FM/TBM' and 'FM' domains are categorized as 'FM' domains in this analysis.

Target	Type	IDs
Domain	TBM	T1024-D1,T1024-D2,T1025-D1,T1026-D1,T1028-D1,T1030-D1,T1030-D2,T1032-D1, T1034-D1,T1036s1-D1,T1045s1-D1,T1045s2-D1,T1046s2-D1,T1047s2-D2,T1050-D1, T1050-D2,T1050-D3,T1052-D1,T1052-D2,T1054-D1,T1056-D1,T1057-D1,T1058-D2, T1060s2-D1,T1060s3-D1,T1061-D3,T1065s1-D1,T1067-D1,T1068-D1,T1070-D2, T1070-D3,T1070-D4,T1073-D1,T1076-D1,T1078-D1,T1079-D1,T1083-D1,T1084-D1, T1087-D1,T1089-D1,T1091-D1,T1091-D2,T1091-D3,T1091-D4,T1092-D1,T1092-D2, T1093-D2,T1094-D1,T1095-D1,T1099-D1,T1100-D1,T1100-D2,T1101-D1,T1101-D2
	FM	T1027-D1,T1029-D1,T1031-D1,T1033-D1,T1035-D1,T1037-D1,T1038-D1,T1038-D2, T1039-D1,T1040-D1,T1041-D1,T1042-D1,T1043-D1,T1046s1-D1,T1047s1-D1, T1047s2-D1,T1047s2-D3,T1049-D1,T1052-D3,T1053-D1,T1053-D2,T1055-D1, T1058-D1,T1061-D1,T1061-D2,T1064-D1,T1065s2-D1,T1070-D1,T1074-D1, T1080-D1,T1082-D1,T1090-D1,T1093-D1,T1093-D3,T1094-D2,T1096-D1,T1096-D2
Full-length		T1024,T1025,T1026,T1027,T1028,T1029,T1030,T1031,T1032,T1033,T1034,T1035, T1036s1,T1037,T1038,T1039,T1040,T1041,T1042,T1043,T1045s1,T1045s2,T1046s1, T1046s2,T1047s1,T1047s2,T1049,T1050,T1052,T1053,T1054,T1055,T1056,T1057, T1058,T1060s2,T1060s3,T1061,T1064,T1065s1,T1065s2,T1067,T1068,T1070,T1073, T1074,T1076,T1078,T1079,T1080,T1082,T1083,T1084,T1087,T1089,T1090,T1091, T1092,T1093,T1094,T1095,T1096,T1099,T1100,T1101

References

1. Zheng, W.; Wuyun, Q.; Li, Y.; Mortuza, S. M.; Zhang, C.; Pearce, R.; Ruan, J.; Zhang, Y., Detecting distant-homology protein structures by aligning deep neural-network based contact maps. *PLOS Computational Biology* **2019,** 15, (10), e1007411.
2. Llinares-López, F.; Berthet, Q.; Blondel, M.; Teboul, O.; Vert, J.-P., Deep embedding and alignment of protein sequences. *Nature Methods* **2023,** 20, (1), 104-111.
3. Bhattacharya, S.; Roche, R.; Moussad, B.; Bhattacharya, D., DisCovER: distance- and orientation-based covariational threading for weakly homologous proteins. *Proteins: Structure, Function, and Bioinformatics* **2022,** 90, (2), 579-588.
4. Pantolini, L.; Studer, G.; Pereira, J.; Durairaj, J.; Tauriello, G.; Schwede, T., Embedding-based alignment: combining protein language models with dynamic programming alignment to detect structural similarities in the twilight-zone. *Bioinformatics* **2024,** 40, (1), btad786.
5. Buchan, D. W. A.; Jones, D. T., EigenTHREADER: analogous protein fold recognition by efficient contact map threading. *Bioinformatics* **2017,** 33, (17), 2684-2690.
6. Xu, D.; Jaroszewski, L.; Li, Z.; Godzik, A., FFAS-3D: improving fold recognition by including optimized structural features and template re-ranking. *Bioinformatics* **2014,** 30, (5), 660-667.
7. Meier, A.; Söding, J., Automatic Prediction of Protein 3D Structures by Probabilistic Multi-template Homology Modeling. *PLOS Computational Biology* **2015,** 11, (10), e1004343.
8. Söding, J., Protein homology detection by HMM–HMM comparison. *Bioinformatics* **2005,** 21, (7), 951-960.
9. Zheng, W.; Zhang, C.; Bell, E. W.; Zhang, Y., I-TASSER gateway: A protein structure and function prediction server powered by XSEDE. *Future generations computer systems : FGCS* **2019,** 99, 73-85.
10. Yang, J.; Zhang, Y., I-TASSER server: new development for protein structure and function predictions. *Nucleic Acids Research* **2015,** 43, (W1), W174-W181.
11. Zhang, Y., Template-based modeling and free modeling by I-TASSER in CASP7. *Proteins: Structure, Function, and Bioinformatics* **2007,** 69, (S8), 108-117.
12. Zheng, W.; Wuyun, Q.; Zhou, X.; Li, Y.; Freddolino, P. L.; Zhang, Y., LOMETS3: integrating deep learning and profile alignment for advanced protein template recognition and function annotation. *Nucleic Acids Research* **2022,** 50, (W1), W454-W464.
13. Zheng, W.; Zhang, C.; Wuyun, Q.; Pearce, R.; Li, Y.; Zhang, Y., LOMETS2: improved meta-threading server for fold-recognition and structure-based function annotation for distant-homology proteins. *Nucleic Acids Research* **2019,** 47, (W1), W429-W436.
14. Wu, S.; Zhang, Y., LOMETS: A local meta-threading-server for protein structure prediction. *Nucleic Acids Research* **2007,** 35, (10), 3375-3382.
15. Ovchinnikov, S.; Park, H.; Varghese, N.; Huang, P.-S.; Pavlopoulos, G. A.; Kim, D. E.; Kamisetty, H.; Kyrpides, N. C.; Baker, D., Protein structure determination using metagenome sequence data. *Science* **2017,** 355, (6322), 294-298.
16. Šali, A.; Blundell, T. L., Comparative Protein Modelling by Satisfaction of Spatial Restraints. *Journal of Molecular Biology* **1993,** 234, (3), 779-815.
17. Ma, J.; Wang, S.; Wang, Z.; Xu, J., MRFalign: Protein Homology Detection through Alignment of Markov Random Fields. *PLOS Computational Biology* **2014,** 10, (3), e1003500.

18. Cheng, J.; Li, J.; Wang, Z.; Eickholt, J.; Deng, X., The MULTICOM toolbox for protein structure prediction. *BMC Bioinformatics* **2012,** 13, (1), 65.
19. Cheng, J., A multi-template combination algorithm for protein comparative modeling. *BMC Structural Biology* **2008,** 8, (1), 18.
20. Wu, S.; Zhang, Y., MUSTER: Improving protein sequence profile–profile alignments by using multiple sources of structure information. *Proteins: Structure, Function, and Bioinformatics* **2008,** 72, (2), 547-556.
21. Kelley, L. A.; Mezulis, S.; Yates, C. M.; Wass, M. N.; Sternberg, M. J. E., The Phyre2 web portal for protein modeling, prediction and analysis. *Nature Protocols* **2015,** 10, (6), 845-858.
22. Kaminski, K.; Ludwiczak, J.; Pawlicki, K.; Alva, V.; Dunin-Horkawicz, S., pLM-BLAST: distant homology detection based on direct comparison of sequence representations from protein language models. *Bioinformatics* **2023,** 39, (10), btad579.
23. Källberg, M.; Wang, H.; Wang, S.; Peng, J.; Wang, Z.; Lu, H.; Xu, J., Template-based protein structure modeling using the RaptorX web server. *Nature Protocols* **2012,** 7, (8), 1511-1522.
24. Peng, J.; Xu, J., Raptorx: Exploiting structure information for protein alignment by statistical inference. *Proteins: Structure, Function, and Bioinformatics* **2011,** 79, (S10), 161-171.
25. Song, Y.; DiMaio, F.; Wang, Ray Y.-R.; Kim, D.; Miles, C.; Brunette, T. J.; Thompson, J.; Baker, D., High-Resolution Comparative Modeling with RosettaCM. *Structure* **2013,** 21, (10), 1735-1742.
26. Gao, M.; Skolnick, J., A novel sequence alignment algorithm based on deep learning of the protein folding code. *Bioinformatics* **2021,** 37, (4), 490-496.
27. Yang, Y.; Faraggi, E.; Zhao, H.; Zhou, Y., Improving protein fold recognition and template-based modeling by employing probabilistic-based matching between predicted one-dimensional structural properties of query and corresponding native properties of templates. *Bioinformatics* **2011,** 27, (15), 2076-2082.
28. Zhang, H.; Shen, Y., Template-based prediction of protein structure with deep learning. *BMC Genomics* **2020,** 21, (11), 878.
29. Jones, D. T., Predicting novel protein folds by using FRAGFOLD. *Proteins: Structure, Function, and Bioinformatics* **2001,** 45, (S5), 127-132.
30. Xu, D.; Zhang, Y., Ab initio protein structure assembly using continuous structure fragments and optimized knowledge-based force field. *Proteins: Structure, Function, and Bioinformatics* **2012,** 80, (7), 1715-1735.
31. Simons, K. T.; Kooperberg, C.; Huang, E.; Baker, D., Assembly of protein tertiary structures from fragments with similar local sequences using simulated annealing and bayesian scoring functions11Edited by F. E. Cohen. *Journal of Molecular Biology* **1997,** 268, (1), 209-225.
32. Rohl, C. A.; Strauss, C. E. M.; Misura, K. M. S.; Baker, D., Protein Structure Prediction Using Rosetta. In *Methods in Enzymology*, Academic Press: 2004; Vol. 383, pp 66-93.
33. Zheng, W.; Zhang, C.; Li, Y.; Pearce, R.; Bell, E. W.; Zhang, Y., Folding non-homologous proteins by coupling deep-learning contact maps with I-TASSER assembly simulations. *Cell reports methods* **2021,** 1, (3).
34. Adhikari, B.; Cheng, J., CONFOLD2: improved contact-driven ab initio protein structure modeling. *BMC Bioinformatics* **2018,** 19, (1), 22.

35. Mortuza, S. M.; Zheng, W.; Zhang, C.; Li, Y.; Pearce, R.; Zhang, Y., Improving fragment-based ab initio protein structure assembly using low-accuracy contact-map predictions. *Nature Communications* **2021,** 12, (1), 5011.
36. Wang, S.; Sun, S.; Li, Z.; Zhang, R.; Xu, J., Accurate De Novo Prediction of Protein Contact Map by Ultra-Deep Learning Model. *PLOS Computational Biology* **2017,** 13, (1), e1005324.
37. Pearce, R.; Li, Y.; Omenn, G. S.; Zhang, Y., Fast and accurate Ab Initio Protein structure prediction using deep learning potentials. *PLOS Computational Biology* **2022,** 18, (9), e1010539.
38. Li, Y.; Zhang, C.; Yu, D.-J.; Zhang, Y., Deep learning geometrical potential for high-accuracy ab initio protein structure prediction. *iScience* **2022,** 25, (6).
39. Zhu, J.; Wang, S.; Bu, D.; Xu, J., Protein threading using residue co-variation and deep learning. *Bioinformatics* **2018,** 34, (13), i263-i273.
40. Zheng, W.; Wuyun, Q.; Freddolino, P. L.; Zhang, Y., Integrating deep learning, threading alignments, and a multi-MSA strategy for high-quality protein monomer and complex structure prediction in CASP15. *Proteins: Structure, Function, and Bioinformatics* **2023,** 91, (12), 1684-1703.
41. Zheng, W.; Li, Y.; Zhang, C.; Zhou, X.; Pearce, R.; Bell, E. W.; Huang, X.; Zhang, Y., Protein structure prediction using deep learning distance and hydrogen-bonding restraints in CASP14. *Proteins: Structure, Function, and Bioinformatics* **2021,** 89, (12), 1734-1751.
42. Greener, J. G.; Kandathil, S. M.; Jones, D. T., Deep learning extends de novo protein modelling coverage of genomes using iteratively predicted structural constraints. *Nature Communications* **2019,** 10, (1), 3977.
43. Shen, T.; Wu, J.; Lan, H.; Zheng, L.; Pei, J.; Wang, S.; Liu, W.; Huang, J., When homologous sequences meet structural decoys: Accurate contact prediction by tFold in CASP14—(tFold for CASP14 contact prediction). *Proteins: Structure, Function, and Bioinformatics* **2021,** 89, (12), 1901-1910.
44. Yang, J.; Anishchenko, I.; Park, H.; Peng, Z.; Ovchinnikov, S.; Baker, D., Improved protein structure prediction using predicted interresidue orientations. *Proceedings of the National Academy of Sciences* **2020,** 117, (3), 1496-1503.
45. Du, Z.; Su, H.; Wang, W.; Ye, L.; Wei, H.; Peng, Z.; Anishchenko, I.; Baker, D.; Yang, J., The trRosetta server for fast and accurate protein structure prediction. *Nature Protocols* **2021,** 16, (12), 5634-5651.
46. Jumper, J.; Evans, R.; Pritzel, A.; Green, T.; Figurnov, M.; Ronneberger, O.; Tunyasuvunakool, K.; Bates, R.; Žídek, A.; Potapenko, A.; Bridgland, A.; Meyer, C.; Kohl, S. A. A.; Ballard, A. J.; Cowie, A.; Romera-Paredes, B.; Nikolov, S.; Jain, R.; Adler, J.; Back, T.; Petersen, S.; Reiman, D.; Clancy, E.; Zielinski, M.; Steinegger, M.; Pacholska, M.; Berghammer, T.; Bodenstein, S.; Silver, D.; Vinyals, O.; Senior, A. W.; Kavukcuoglu, K.; Kohli, P.; Hassabis, D., Highly accurate protein structure prediction with AlphaFold. *Nature* **2021,** 596, (7873), 583-589.
47. Mirdita, M.; Schütze, K.; Moriwaki, Y.; Heo, L.; Ovchinnikov, S.; Steinegger, M., ColabFold: making protein folding accessible to all. *Nature Methods* **2022,** 19, (6), 679-682.
48. Cheng, S.; Zhao, X.; Lu, G.; Fang, J.; Yu, Z.; Zheng, T.; Wu, R.; Zhang, X.; Peng, J.; You, Y., FastFold: Reducing AlphaFold Training Time from 11 Days to 67 Hours. *arxiv:2203.00854[cs.LG,cs.AI,cs.DC,q-bio.QM]* **2022**.

49. Wang, G.; Fang, X.; Wu, Z.; Liu, Y.; Xue, Y.; Xiang, Y.; Yu, D.; Wang, F.; Ma, Y., HelixFold: An Efficient Implementation of AlphaFold2 using PaddlePaddl e. *arxiv:2207.05477[cs.DC,cs.LG,q-bio.BM]* **2022**.
50. Liu, S.; Zhang, J.; Chu, H.; Wang, M.; Xue, B.; Ni, N.; Yu, J.; Xie, Y.; Chen, Z.; Chen, M.; Liu, Y.; Patra, P.; Xu, F.; Chen, J.; Wang, Z.; Yang, L.; Yu, F.; Chen, L.; Gao, Y. Q., PSP: Million-level Protein Sequence Dataset for Protein Structure Prediction. *arxiv:2206.12240[q-bio.BM,cs.LG]* **2022**.
51. Gustaf, A.; Nazim, B.; Christina, F.; Sachin, K.; Qinghui, X.; William, G.; Timothy, J. O. D.; Daniel, B.; Ian, F.; Niccolò, Z.; Bo, Z.; Arkadiusz, N.; Bei, W.; Marta, M. S.-D.; Shang, Z.; Adegoke, O.; Murat Efe, G.; Stella, B.; Andrew, M. W.; Stephen, R.; Pablo Ribalta, L.; Lucas, N.; Brian, W.; Yih-En Andrew, B.; Peter, K. S.; Emad, M.; Zhao, Z.; Richard, B.; Mohammed, A., OpenFold: Retraining AlphaFold2 yields new insights into its learning mechanisms and capacity for generalization. *bioRxiv* **2023**, 2022.11.20.517210.
52. Baek, M.; DiMaio, F.; Anishchenko, I.; Dauparas, J.; Ovchinnikov, S.; Lee, G. R.; Wang, J.; Cong, Q.; Kinch, L. N.; Schaeffer, R. D.; Millán, C.; Park, H.; Adams, C.; Glassman, C. R.; DeGiovanni, A.; Pereira, J. H.; Rodrigues, A. V.; van Dijk, A. A.; Ebrecht, A. C.; Opperman, D. J.; Sagmeister, T.; Buhlheller, C.; Pavkov-Keller, T.; Rathinaswamy, M. K.; Dalwadi, U.; Yip, C. K.; Burke, J. E.; Garcia, K. C.; Grishin, N. V.; Adams, P. D.; Read, R. J.; Baker, D., Accurate prediction of protein structures and interactions using a three-track neural network. *Science* **2021,** 373, (6557), 871-876.
53. Ziyao, L.; Xuyang, L.; Weijie, C.; Fan, S.; Hangrui, B.; Guolin, K.; Linfeng, Z., Uni-Fold: An Open-Source Platform for Developing Protein Folding Models beyond AlphaFold. *bioRxiv* **2022**, 2022.08.04.502811.
54. Konstantin, W.; Michael, H.; Martin, S.; Burkhard, R., Ultra-fast protein structure prediction to capture effects of sequence variation in mutation movies. *bioRxiv* **2022**, 2022.11.14.516473.
55. Lin, Z.; Akin, H.; Rao, R.; Hie, B.; Zhu, Z.; Lu, W.; Smetanin, N.; Verkuil, R.; Kabeli, O.; Shmueli, Y.; dos Santos Costa, A.; Fazel-Zarandi, M.; Sercu, T.; Candido, S.; Rives, A., Evolutionary-scale prediction of atomic-level protein structure with a language model. *Science* **2023,** 379, (6637), 1123-1130.
56. Fang, X.; Wang, F.; Liu, L.; He, J.; Lin, D.; Xiang, Y.; Zhu, K.; Zhang, X.; Wu, H.; Li, H.; Song, L., A method for multiple-sequence-alignment-free protein structure prediction using a protein language model. *Nature Machine Intelligence* **2023,** 5, (10), 1087-1096.
57. Ruffolo, J. A.; Chu, L.-S.; Mahajan, S. P.; Gray, J. J., Fast, accurate antibody structure prediction from deep learning on massive set of natural antibodies. *Nature Communications* **2023,** 14, (1), 2389.
58. Ruidong, W.; Fan, D.; Rui, W.; Rui, S.; Xiwen, Z.; Shitong, L.; Chenpeng, S.; Zuofan, W.; Qi, X.; Bonnie, B.; Jianzhu, M.; Jian, P., High-resolution de novo structure prediction from primary sequence. *bioRxiv* **2022**, 2022.07.21.500999.
59. Xiaoyang, J.; Fandi, W.; Jinbo, X., RaptorX-Single: single-sequence protein structure prediction by integrating protein language models. *bioRxiv* **2023**, 2023.04.24.538081.
60. Wang, W.; Peng, Z.; Yang, J., Single-sequence protein structure prediction using supervised transformer protein language models. *Nature Computational Science* **2022,** 2, (12), 804-814.

61. Xu, D.; Jaroszewski, L.; Li, Z.; Godzik, A., AIDA: ab initio domain assembly for automated multi-domain protein structure prediction and domain–domain interaction prediction. *Bioinformatics* **2015,** 31, (13), 2098-2105.
62. Xia, Y.; Zhao, K.; Liu, D.; Zhou, X.; Zhang, G., Multi-domain and complex protein structure prediction using inter-domain interactions from deep learning. *Communications Biology* **2023,** 6, (1), 1221.
63. Zhou, X.; Peng, C.; Zheng, W.; Li, Y.; Zhang, G.; Zhang, Y., DEMO2: Assemble multi-domain protein structures by coupling analogous template alignments with deep-learning inter-domain restraint prediction. *Nucleic Acids Research* **2022,** 50, (W1), W235-W245.
64. Zhou, X.; Hu, J.; Zhang, C.; Zhang, G.; Zhang, Y., Assembling multidomain protein structures through analogous global structural alignments. **2019,** 116, (32), 15930-15938.
65. Zhu, H.-T.; Xia, Y.-H.; Zhang, G.-J., E2EDA: Protein Domain Assembly Based on End-to-End Deep Learning. *Journal of Chemical Information and Modeling* **2023,** 63, (20), 6451-6461.
66. Zhou, X.; Zheng, W.; Li, Y.; Pearce, R.; Zhang, C.; Bell, E. W.; Zhang, G.; Zhang, Y., I-TASSER-MTD: a deep-learning-based platform for multi-domain protein structure and function prediction. *Nature Protocols* **2022,** 17, (10), 2326-2353.
67. Peng, C.-X.; Zhou, X.-G.; Xia, Y.-H.; Liu, J.; Hou, M.-H.; Zhang, G.-J., Structural analogue-based protein structure domain assembly assisted by deep learning. *Bioinformatics* **2022,** 38, (19), 4513-4521.
